# Dairy Cows Produce Less Milk and Modify Their Behaviour during the Transition between Tie-Stall to Free-Stall

**DOI:** 10.3390/ani7030016

**Published:** 2017-03-03

**Authors:** Jan Broucek, Michal Uhrincat, Stefan Mihina, Miloslav Soch, Andrea Mrekajova, Anton Hanus

**Affiliations:** 1 Research Institute of Animal Production Nitra, 951 41 Luzianky, Slovakia; uhrincat@vuzv.sk (M.U.); mrekajova@vuzv.sk (A.M.); hanus@vuzv.sk (A.H.); 2 Faculty of Engineering, Slovak Agriculture University Nitra, 949 01 Nitra, Slovakia; stefan.mihina@gmail.com; 3 Faculty of Agriculture, University of South Bohemia Ceske Budejovice, 370 05 Ceske Budejovice, Czech Republic; soch@zf.jcu.cz

**Keywords:** dairy cow, milk yield, behaviour, housing, milking

## Abstract

**Simple Summary:**

The purpose of this study was to evaluate the influence of moving cows from the barn with stanchion-stall housing to free-stall housing on their behaviour and production. Cows lay down up to ten hours after removing. The cows in their second lactation and open cows tended to lie sooner after removing than cows in their first lactation and pregnant cows. The times of total lying and rumination were increasing from the first day to the tenth day after removing. Cows produced 23.3% less milk at the first day following the transfer than at the last day prior to moving (23.76 ± 7.20 kg vs. 30.97 ± 7.26 kg, *p* < 0.001). Loss of milk was gradually reduced and on the 14th day, cows achieved maximum production. The difference was found in milk losses due to the shift between cows in first and second lactation.

**Abstract:**

Transfer of cattle to an unknown barn may result in a reduction in its welfare. Housing and management practices can result in signs of stress that include a long-term suppression of milk efficiency. The purpose of this study was to evaluate the influence of moving cows from the stanchion-stall housing to free-stall housing on their behaviour and production. The Holstein cows were moved into the new facility with free-stall housing from the old barn with stanchion-stall housing. Cows lay down up to ten hours (596.3 ± 282.7 min) after removing. The cows in their second lactation and open cows tended to lie sooner after removing than cows in their first lactation and pregnant cows. The times of total lying and rumination were increasing from the first day to the tenth day after removing (23.76 ± 7.20 kg vs. 30.97 ± 7.26 kg, *p* < 0.001). Cows produced 23.3% less milk at the first day following the transfer than at the last day prior to moving (*p* < 0.001). Loss of milk was gradually reduced and maximum production was achieved on the 14th day. The difference was found in milk losses due to the shift between cows on the first and second lactation (*p* < 0.01). The results of this study suggest that removing from the tie-stall barn with a pipeline milking system into the barn with free-stall housing and a milking parlour caused a decline in the cows’ milk production. However, when the cows are moved to a better environment, they rapidly adapt to the change.

## 1. Introduction

Many dairy buildings are relatively old and cow size has increased progressively in past decades [1]. Tethering cows restricts their freedom of movement [2]. Therefore, a number of farms are currently changing from individual housing to group housing. However, the relocation process has been implicated as one of the major aversions for received cattle [3]. The welfare impairment associated with removing and arrival at a new facility can be one of the most stressful situations an animal experiences and can cause a number of physiological and behavioural changes including altered hormones, metabolites of energy and protein metabolism, and also changes in milk production [4,5,6]. During relocation, cattle are subjected to noise, strange surroundings, odours and companions, overcrowding or sometimes isolation, hot or cold conditions, and a change of feed [7,8,9]. All of these factors contribute to stress and potential performance losses.

There is limited information describing changes in production associated with relocation of lactating dairy cows. Soch et al. [10] recorded decrease of milk yield from 19.0 kg before treatment on 10.2 kg in the first day after moving from stanchion-stall to free-stall housing. Norell and Appleman [11] declared that there was no effect on yearly milk production the first year following relocation. After changing from stanchion-stall to free-stall housing, cows produced nearly 200 kg less 3.5% fat-corrected milk than stanchion herds. However, yearly differences in milk yield did not occur between the changes of the four housing systems and control herds significantly. Brakel and Leis [12] observed that the milk yield of regrouped cows decreased by 3% on the first day following regrouping. Phillips and Rind [13] found that milk yield of mixed cows was 3% less in the first week than cows in the unmixed groups, and 1% less in the sixth week after mixing. The reduction in milk yield was similar for first- and second-lactation cows.

Dairy cows are relatively adaptable to a wide range of environments. Various criteria have been proposed to identify inappropriate management and housing conditions for them [14,15,16]. Some researchers have placed emphasis on criteria of well-being [17]. Cow comfort is widely recognized as an important effect on propensity to produce milk [18,19,20]. When a cow is not standing comfortably in the milking parlour, she can be stressed. Stress factors in the milking parlour include small stands, inconvenient hygiene, inappropriate microclimate, and insufficient floors. Cow reactions to parlour stress include not entering the parlour voluntarily, kicking off the milking cluster, defecating in the parlour or refusing milk let-down [8,18]. A modern dairy facility requires quality design, construction and ultimately management to provide a cow-centered living space. The novelty of this study is examining the impact of the manner of milking and parlour change on cows.

The purpose of this study was to evaluate the influence of the acute stress associated with the environment transition when relocating dairy cows to different housing on their milk production and maintenance behaviour. We hypothesized that treatment would affect time spent lying, standing and ruminating, and also milk yield.

## 2. Materials and Methods

### 2.1. Animals

In order to evaluate the adaptability after relocation to a new housing type, 41 Holstein cows on first and second lactation with the average age of 1244 ± 113 days (from 1080 to 1380 days) and live body weight of 558 ± 45 kg were observed. Eighteen cows were in their first lactation and 23 in their second lactation. Cows were kept in two pens (movement area 7.4 m^2^ per animal, concrete alleys 2.6 m wide) with free stalls (1.15 × 2.0 m). There were 21 cows in Pen 1, and 20 cows in Pen 2. The groups were balanced according to parity. There were 9 first-parity cows and 12 second-parity cows in Pen 1 (1284 ± 107 days, 559 ± 45 kg), and 9 first-parity cows and 11 second-parity cows in Pen 2 (1200 ± 102 days, 556 ± 47 kg). Feed was available throughout the 24 h period, except during milking. The average day in milk and days pregnant for all cows were 203 ± 135 days and 52 ± 75 days. In total, there were 21 cows pregnant and 20 open. Eleven cows were pregnant and 10 open in Pen 1. Ten heads from both categories were in Pen 2 (10 pregnant and 10 open). The mean stages of gravidity were 101 ± 69 days in the first-lactation group and 37 ± 53 days in second-lactation cows.

### 2.2. Treatment and Housing

The cows were moved into the new facility with free-stall housing from the old barn with stanchion-stall housing. The average daily air temperature and relative humidity in the housing facility were 14.4 ± 1.52 °C and 79.1% ± 3.15%, respectively, during the last week period.

On the morning of the relocation day, farm employees led cows to a rebuilt facility with free-stall housing, a distance of 120 m. All cows were moved to the new housing together, at the same time (at once). The mean daily air temperature and relative humidity in the housing facility were 14.6 ± 1.6 °C and 81.3% ± 3.0%, respectively, during the experimental period of 25 days. These parameters were continuously recorded using data loggers.

Before relocation, cows were housed in stanchion-stall housing in the old facility (with low arch chain ties, dimensions of platform 1.125 m width, 1.65 m length). The new facility was group housing with free stalls, which consists of a solid concrete floor sloping towards a drain in the middle. Manure was removed with a scraper. Cows were kept in two pens (movement area 7.4 m^2^ per animal, concrete alleys 2.6 m wide). Free stalls (1.15 × 2.0 m) were concrete-bedded with straw mattresses above the concrete floor covered lightly with sawdust. Both pens were continuously illuminated throughout the experiment.

The experimental period lasted for 25 days (during the months of November–December 2012). The study was performed in Nitra, Slovakia. The local climate is Cfb, according to the Köppen climate classification. The “C” climate is defined as one with the coldest month’s average temperature below 18 °C and above −3 °C; the warmest month’s average temperature is above 10 °C. The letter “f” represents a climate where no dry season occurs, the “b” the warmest month < 22 °C, but at least 4 months > 10 °C [21].

### 2.3. Milking

Cows in the stanchion-stall housing were milked with a pipeline milking system with the vacuum level of 50 kPa (standard high-line system), pulsation rate 57 cycles per min, and pulsation ratio 60:40. The last two individual milk yields were recorded during the evening and morning milking before shifting (05:00 and 16:00 h). Individual milk yields were weighted as the amount of milk from the milk can (pail) on an electronic scale.

After removing in the free-stall housing facility, the cows were milked in a double-five herringbone design (with vacuum level 50 kPa, pulsation rate 55 cycles per minute and pulsation ratio 60:40) and both side-opening at the same time as before removing. The parameters of both milking systems were similar.

Individual milk yields were measured electronically. Each electronic milk meter was checked the last day before starting the trial and then two times per each week in order to calculate its deviation level. This was done by comparison of the amount of milk weighed on an electronic scale. All electronic meters had a tolerance level to within 3%. The cows were not habituated to milking in a free-stall barn.

The first milking after removing was at the evening milking, and the second one the next morning. Individual milk yields (25 days) were recorded electronically at each morning and evening milking. Individual milk yield per day was calculated as the sum of the evening and morning yields. The decrease in the amount of milk in the first day (D01) and the increase in the amount of milk on the 14th day (D141) were calculated.

The cows were milked twice a day at 05:00 and 16:00 after being driven by the herdsman a short distance within the barn to a holding area, which measured 13.5 m × 4.5 m, adjacent to the milking parlour. The time spent by each cow in the holding area before milking varied from 15 to 30 min. Total times of standing during a milking session (i.e., before and after milking in the holding area, standing in the milking parlour) represented in the first, second and tenth days were 118 ± 30 min, 95 ± 22 min, and 100 ± 23 min, respectively. Cows entered the parlour individually once a milking stall was available. Upon exiting the parlour, cows remained in a separate holding area until all other cows in the group were milked. The cows then walked through an alley, passed over a set of scales, and had access to their free-stall pens immediately.

### 2.4. Feeding

The cows were fed a total mixed ration consisting of maize silage (6.72 kg·DM^−1^), alfalfa haylage (6.18 kg·DM^−1^), alfalfa hay (3.22 kg·DM^−1^), barley straw (0.38 kg·DM^−1^), brewer’s grain (0.38 kg·DM^−1^), sugar-beet pulp (0.57 kg·DM^−1^), and concentrate mixture for high-yielding cows (1.73 kg·DM^−1^) throughout the study. Feed ration contained 19.2 kg DM, 131.0 MJ NEL, 1.84 kg PDI, 2.89 kg of crude protein and 3.41 kg of crude fibre. Diet was the same from one week prior to relocation through four weeks following the move. Following relocation, all cows had daily feed prepared in troughs. Subsequently, they were fed once daily at 10:00 h. The total mixed diet was administered to troughs in the new cubicle barn by a feeding wagon. Feeding was allowed throughout the 24 h period, except during milking. Feed bunks were located centrally to the free-stall pens; raised 0.68 m above ground and 0.7 m of feeding space. Cows did not receive concentrates separately. Automatic watering troughs were located next to feed bunks and at the end of free-stall pens.

### 2.5. Ethological Observations

Cows were observed for three 24 h periods (first, second and tenth day) after moving into the new facility with cubicle housing, and behavioural observations were recorded at 10 min intervals (from 10:00 h). Behavioural data were obtained by video observations and electronic measurements. The barn was equipped with video cameras for continuous filming of the cows’ activities. There were computer technics and software for evaluation (cameras: Samsung SCB-3000P, HDD recorder Versatile H.264 DVR), the Observer XT, Noldus (software on transmitten behavioural activities into numerical data). During the 24 h of observation, two observers were always present (for controlling); one person for each pen. They changed after 12 h; four observers were totally (they were mostly the authors of the manuscript), highly experienced in the evaluation of welfare and health. They received directives, of course, one day before the start of the experiment; and also conducted training. Data were obtained from the video, done by the author of the article.

Thus, the lying-down behaviour of cows can be an important criterion in the assessment of comfort after relocation, which we used in the research and comprehensive assessment of laterality, including lying on the left and right sides. Time spent lying (on the left and right side), standing (with or without movement, including time spent in the milking parlour), feeding, ruminating (ruminating while standing plus ruminating while lying), as well as the number of activity bouts were calculated. Based on this data, activity latencies and episodes were calculated.

The latency for each cow to the initiation of defined behaviour after relocation was determined. Latency time for total lying is first time lying down, regardless of which side the cow lay on. Latency for lying on the left or right side was calculated as first time lying down on the observed side (time between the onset of a stimulus and the initiation of the response, or recording how long it takes the cow to initiate the behaviour after a particular event occurs). Duration of behaviour episodes were summarized as a continuous series of records of the same activity lengths after relocation.

### 2.6. Statistical Evaluation

The data were analysed using a General Linear Model analysis of variance (ANOVA) as repeated measures by the statistical package STATISTIX [22]. The normality of data distribution was evaluated by the Wilk–Shapiro/Rankin Plot procedure. All data conformed to a normal distribution. Significant differences between groups were tested by Comparisons of Mean Ranks. Differences among means were tested by Bonferroni’s method. Values are expressed as means ± SD (standard deviation).

The evaluated factors were day (of ethological observation or milk recording), lactation number (first, second), and gestation (pregnant, open). The dependent variables included daily milk yield; times spent lying, lying on the left and right sides, standing, feeding, and ruminating; latencies of lying, lying on the left and right sides, feeding, and ruminating; lengths and numbers of episodes lying, lying on the left and right sides, standing, feeding, and ruminating.

## 3. Results

### 3.1. Maintenance Behaviour

**Figure 1:**
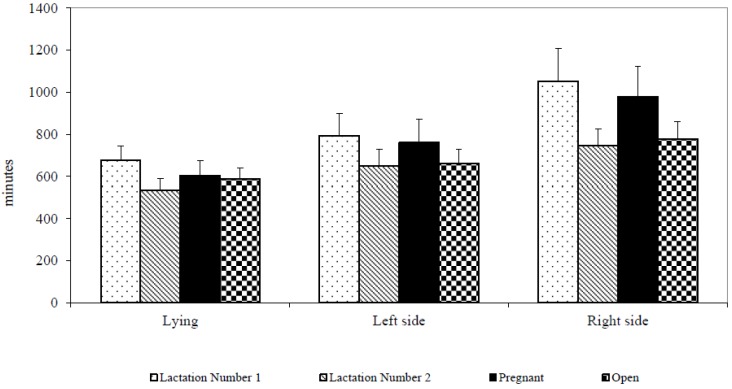
Latency of resting behaviour after removing (minutes).

The observed cows generally lay down up to ten hours (596.3 ± 282.7 min). The cows in their second lactation and open cows lay down sooner after relocation to a new barn than cows in their first lactation and pregnant cows. The biggest differences—but statistically insignificant—were found between first and second lactation in the times of the first lying on the left side (793.3 ± 453.2 min vs. 648.7 ± 382.2 min, df 1,1,40; *p* ≥ 0.05), and lying on the right side (1053.3 ± 663.7 min vs. 745.0 ± 380.0 min, df 1,1,39; *p* ≥ 0.05). Latencies of first lying tended to vary also between pregnant and open cows in the lying on the left side (760.5 ± 504.9 min vs. 661.5 ± 300.5 min, df 1,1,40; *p* ≥ 0.05), and lying on the right side (980.0 ± 654.8 min vs. 777.4 ± 371.0 min, df 1,1,39; *p* ≥ 0.05) (Figure 1).

Dissatisfaction or restlessness of cows is reflected in the frequently changing positions and behaviour activities. This study recorded a trend of non-significantly lower episode numbers of total lying (1.25 ± 0.5 vs. 2.67 ± 1.63; 1.6 ± 0.55 vs. 2.6 ± 1.95), lying on the left (1.33 ± 0.58 vs. 1.71 ± 1.11; 1.5 ± 0.58 vs.1.67 ± 1.21) and right sides (1.0 ± 0.0 vs. 1.4 ± 0.55; 1.0 ± 0.0 vs.1.5 ± 0.58) in first-lactation cows and pregnant cows in comparison to second-lactation cows and open cows.

**Figure 2:**
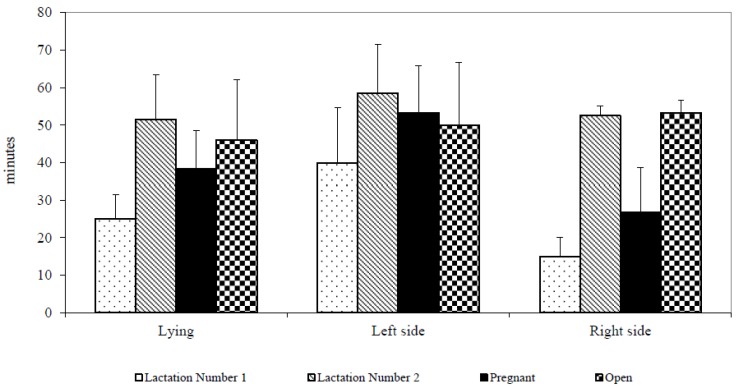
Lengths of resting episode after removing (minutes).

First-lactation cows had shorter lying episodes on the left side (40.0 ± 29.4 min vs. 58.6 ± 33.9 min; df 1,1,10; *p* < 0.001), and the lying time on the right side (15.0 ± 7.1 min vs. 52.5 ± 5.0 min; df 1,1,5; *p* < 0.001) than second-lactation cows. Pregnant cows exhibited longer duration of the first lying episode on the left side (53.3 ± 30.8 min vs. 50.0 ± 37.4 min; df 1,1,10; *p* < 0.01), and a longer time of ruminating (18.5 ± 14.2 min vs. 15.5 ± 9.4 min; df 1,1,10; *p* < 0.05) than open cows following the relocation (Figure 2).

Large differences were recorded in the evaluation of the behaviour during the first, second and tenth day after the transfer. The times of total lying (336.3 ± 171.1 min; 628.0 ± 181.2 min; 756.1 ± 140.3 min; df 1,122; *p* < 0.001) were increasing from the first day to the tenth day after relocation. The cows lay longer on their left sides during all days. A similar course—but not significant—was found at rumination time (318.0 ± 58.7 min; 325.4 ± 74.1 min; 440.5 ± 77.4 min). The total time of standing was decreasing (1103.7 ± 171.1 min; 811.9 ± 181.2 min; 683.9 ± 140.3; df 1,122; *p* < 0.001) from the first day to the tenth day.

### 3.2. Milk Yield

**Figure 3:**
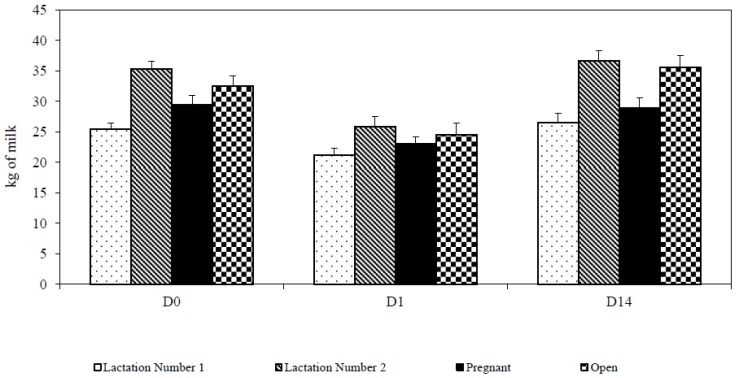
Milk yield after removing (D0 = last day before removing; D1 = first day after removing; D14 = fourteenth day after removing).

Relocation had a large significant negative impact on daily milk yield. Cows produced 7.21 kg milk (23.3%) less at the first day (D1) following the transfer than at the last day prior to moving (D0) (23.76 ± 7.20 kg vs. 30.97 ± 7.26 kg, df 1,1,40; *p* < 0.001); on the second day milk production increased from D1 (27.53 ± 8.09 kg). Loss of milk was gradually reduced and, on the 14th day (D14), achieved maximum production (32.16 ± 8.87 kg), which represented an increase of 8.84 kg (28.5%) of milk yield. The difference between D1 and D14 was also significant (df 1,1,40; *p* = 0.015). A similar course was observed in all evaluated factors (lactation number, pregnancy) (Figure 3).

**Figure 4:**
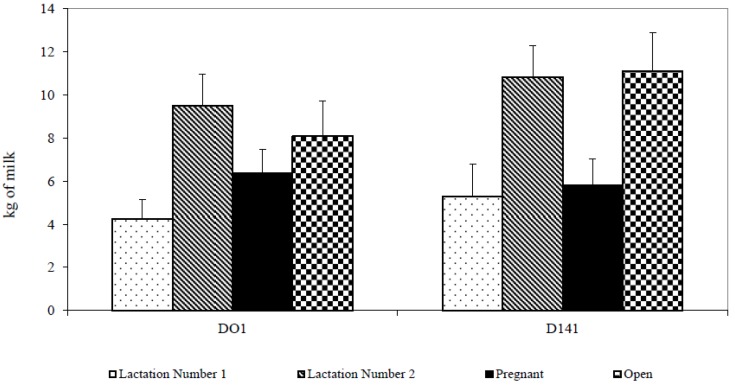
The decrease and increase in milk yield due to the shift of cows (D01 = decrease in the amount of milk on the first day after removing; D141 = increase in the amount of milk on the fourteenth day after removing).

Open cows decreased milk yield immediately after relocating more so than pregnant cows (8.08 ± 7.37 kg vs. 6.38 ± 5.00 kg). Decrease of milk immediately after removing (30.95 ± 7.23 kg vs. 23.77 ± 7.22 kg, df 1,1,40; *p* < 0.01) was significantly lower in first-lactation cows (4.26 ± 3.74 kg vs. 9.51 ± 6.89 kg, *p* < 0.01), but the increase on the 14th day (D141) (23.76 ± 7.20 kg vs. 31.82 ± 8.96 kg, df 1,1,40; *p* < 0.05) was also lower than in second-lactation cows (5.29 ± 6.45 kg vs. 10.83 ± 6.92 kg, df 1,1,40; *p* < 0.05) (Figure 4).

## 4. Discussion

This study was conducted to determine that the factors of day, lactation number, and reproductive status have an effect on behaviour after dairy cows shift from a stanchion-stall barn to a free-stall barn. The central topics to be considered in this paper are the ways in which behaviour and production responses change during unknown environment. Relocation had a negative impact on daily milk yield [10,11]. Therefore, we used the following measures as latency to perform the first rest, eating and ruminating, lengths and number the first maintenance episodes of behaviours. Dissatisfaction or restlessness of cows is reflected in the frequently changing positions and behaviour activities [8,12].

Dairy cattle present few behaviour problems in general probably because farmers have selected docile animals as well as high producers. Farmers tend to cull cows from the herd that do not conform to the system. Dairy cows are docile by nature and only change their behaviour in response to environmental or management changes. The biggest problems that arise with dairy cows concern changes in their management [23]. However, there are many other factors that cause these cows to suffer when adapting. A sudden novel event can be highly stressful to an animal [24,25]. When cows that had formerly been milked and fed simultaneously in a stanchion barn are placed in free stalls and are not fed concentrates when they are milked, they may be dissatisfied and restless [23]. Dairy cows pay greater attention to moving objects than to static ones, but their perception of motion is less fluid than in humans. Therefore, sudden movements by humans may cause anxiety and even panic reactions [24]. Cattle perception ability is also used to characterize individual animal behaviours under farm conditions—especially social behaviour.

After relocation, it is important to find how long it takes dairy cows to calm down and start performing normal patterns of behaviour. After moving, cows spend more time standing. The number of standing cows after relocating depends on the suitability of the barn conditions and the experience of the operator who moves the cows [26]. When unfamiliar cows first meet, fighting often occurs to establish rank. Once hierarchical structure within a group is established, negative interactions become less common except for a limited feed, or preferred lying areas [27,28,29,30].

Cows occasionally manifested a reduced latency to first lying down. Chaplin et al. [31] reported about significant findings when cows showed more lying after deprivation of lying. Lying deprivation can cause a rest disturbance. Krohn and Munksgaard [32] and Bolinger et al. [33] wrote that cows changed their lying position depending on whether or not they were restricted from lying down before.

Second-lactation cows and open cows lay down sooner than cows in their first lactation or pregnant cows. It could be due to multiple causes. According to our opinion, second-lactation cows needed less time to lie down due to a faster adaptation phase; they are older and more experienced. After a housing change, cows could have more interrupted attempts at lying and explore the lying area more prior to lying down. Yet why open cows lie down earlier than pregnant cows is difficult to explain. There is a lack of sources; nobody has probably dealt with this problem except for us. One reason could be that these cows were placed in the social order at a higher level. Evaluating the adaptation of dairy cows to new housing is a significant indicator length of the first episode of specific behaviour activity. First-lactation cows had shorter lying episodes on the left side and the lying time on the right side than second-lactation cows. Pregnant cows exhibited longer duration of the first lying episode on the left side, and a longer time of ruminating than open cows following the relocation. However, neither lactation order nor pregnancy status statistically affected latency to lie down, ruminating or feeding. Similarly, lengths of maintenance activities episodes after removing were not significantly different among treatment groups following relocation. Another possible explanation is that the open cows are higher yielding than those cows early after parturition or pregnant.

We found that after relocation and a housing change, the times of lying increased gradually from the first to the third observation in the tenth day. A similar course was found at rumination time. Relocation and mixing of unfamiliar cows resulted in modification of behaviour immediately following the change. The lying time was reduced; an increase in time spent standing was recorded. However, these modifications were clearly evident only during the first day after moving and a change of housing type. The expected decreased lying time on the day of relocation may have been due to some cows being much less willing to displace others to gain access to a preferred free stall [34], especially primiparous cows, which when first exposed to free stalls in a competitive environment at feeding, may have decreased lying time [35]. However, in this work, the groups were not equally balanced; obtained results can be questionable.

The lying-down behaviour of cows is an important criterion in comfort; therefore, we used comprehensive assessment of laterality, including lying on the left and right sides. Laterality of lying can be an interesting welfare indicator; particularly in assessing changes in health status in dairy cattle [36,37]. Laterality is not random, but is motivated by the amount of rumen fill, slope of the floor, stage of gestation, occupancy of an adjacent stall [38,39].

In this study, the cows lay longer on their left sides during all days. First-lactation cows had shorter lying episodes on the left side, and the lying time on the right side, than second-lactation cows. Pregnant cows exhibited longer duration of the first lying episode on the left side, and a longer time of ruminating than open cows following the relocation. It is very difficult to explain this phenomenon. However, after analyzing the cows according to pregnancy stage, we discovered that mean stages of gravidity were 101 days in the first-lactation group and 37 days in second-lactation cows. Our findings are comparable with the pattern within the literature. Previous studies [38,39] have suggested that cows in later stages of pregnancy tend to lie down more on the left side because the fetus is located mainly on the right side of the body.

Relocation to a new facility did not involve long transport periods or constantly changing surroundings. Therefore, we expected that there would be an increase in behavioural and physiological indicators of stress, but these responses would be short-lived as the cows became acclimated to their new habitat. The welfare of dairy cattle can be maintained and their needs met under a variety of management systems [40]. The coping of cattle with different environment processes certainly involves a change in neuronal and endocrine functions [41,42,43]. Therefore, the relocation process is generally recognized as a stressful event. We have few objective criteria with which to judge the well-being in dairy farms yet, and many of the fundamental questions remain unanswered and often unconsidered [8,17,44]. For another animal that has had no previous experience with being restrained, it may react violently and physiological stress indicators will be high. The stress response is affected by an interaction between the animal’s previous experiences and temperament and other inherited behavioural traits. On the other hand, according to [25], attempts to acclimate older cows to handling have been less successful. This may be due to previous experiences with aversive handling. The findings of Adamczyk et al. [30] indicate that the behaviour of loose-housed cows depends on whether their behavioural needs have been met. Once the animals were provided with a sufficient number of feeding places and ad libitum access to good quality feed, no social behaviours associated with temperament or dominance/submissiveness relationships at the feeding table were observed. Regrouping may also affect the stocking density within the pen, which also affects competitive encounters among cows. Increasing stocking density can increase competition over feed and decrease the time cows spend feeding and lying down.

Cows produced significantly less milk at the first day following the relocation than at the last day before. This important result can be explained on the basis of many aspects. Animals in group housing are often regrouped with unfamiliar herd-mates [34,45,46], and this regrouping can induce stress-related behavioural and physiological reactions. Generally, after relocating into the unknown barn, cows stand only, do not want to eat and lie down, and relax. Rest and rapid food intake are guidelines for preventing a decline in milk following a change of environment [7,13]. Dairy herds require a high level of management to ensure that the health and welfare standards for the cows are met. While removing is part of the relocation process, there are multiple factors such as pathogen exposure, commingling, feed ration changes, handling, and acclimation to a novel environment that contribute to the shipping stress complex generally associated with newly received cattle [15]. It is common to regroup cows during lactation and it is said that this usually does not adversely affect production. However, a reduction in milk production after regrouping has been reported by Hasegawa et al. [47] and this can reach 4% during the first five days [48]. When cows were overstocked, there was increased competition for free stalls. They spent less time lying in the free stalls and more time standing in the alley. Also, rumination time is decreased [26,49]. The relocation process is generally regarded as stressful to cattle and includes both physical and psychological stimuli that can cause detrimental physiological and endocrine changes. These physiological and endocrine changes can often potentiate or alter other physiological, immunological, or endocrine responses. Consequently, the release of cortisol associated with the relocation process can cause cattle to be more susceptible to disease through immunosuppression [15,39]. Sevi et al. [50] studied the stressfulness in sheep. They found that regrouping and relocation induced short-term effects on production performance. Moving and mixing were found to cause increased cortisol secretion and to have a slight and short-term effect on the productive traits of lactating cows. Former trials have shown that regrouping has no or very slight impact on milk yield in dairy cattle [12,39], except when this practice involves animals that are presumably more susceptible to stress, such as primiparous cows of low social rank [47].

As stated by Grandin [3] and Broom [17], animal housing and management systems are the most important causes of poor welfare, which impairs the production. These responses would vary with the environmental terms but are also considerably affected by the metabolic pressures on the individual [6,15]. Environmental conditions that elicit physiological coping responses in animals cause deterioration of well-being and slow adaptation of cattle [38]. Changes in the milking parlour can also affect cow behaviour [51]. Being milked in an unfamiliar environment can cause the inhibition of milk ejection.

In the present study, relocation caused prolonged stress, but cows quickly adapted to the new facility. Milk yield was gradually increased and, on the 14th day, reached maximum production. Why did it not last longer? We can explain this through better conditions in the new barn and better well-being in free-stall housing. The freedom of movement in tie stalls is restricted in a way that unnecessary suffering is caused [2]. Also, Holroyd et al. [52] found that growth decrease of beef cattle was recovered after five days. Phillips and Rind [13] reviewed more papers and highlight the various milk reductions after cows mixing. The social relationship between cows is usually established within a few days after grouping [13,52,53]. During these first days, aggressive encounters are common. When the dominance relationships are established, conflicts are often resolved through threats and avoidance. It seems that the consequences of relocation have no long-lasting effect in dairy cows.

In this study, cows on second lactation yielded more than first-lactation cows, not only prior to removing, but also following the move. However, second-lactation cows had higher declines in milk yield than first-lactation cows. This can be explained by their yielding. The response of high-yielding cows is usually greater than low-yielding cows. However, the drop did not last too long. Cows in free-stall housing have greater freedom for movement and exercise, and have more opportunity to improve production. Also, open cows decreased milk yield immediately relocating more than pregnant cows. Open cows are much more likely to be sensitive. They may not yet be adapted after a difficult delivery, but, generally, it depends on the stage of lactation or pregnancy [54,55]. The impact of pregnancy on milk yield depends on lactation stage. The effect is higher in mid-lactation than in late-lactation [54]. A significant effect of pregnancy on milk yield is usually observed from the fifth month of gestation onwards [54,55,56].

It should be noted again that the first-lactation cows and pregnant cows could be included in the lower level of the social hierarchy. The behavioural studies by more authors [57,58,59] showed that the low-ranked cows had a more efficient eating pattern with less time spent in an eating area and fewer visits made to the feed troughs. However, dominance becomes important only when there is a very limited amount of food for which to compete [38,48].

We studied the available literature, but the effect of pregnancy on the social behaviour or hierarchy of dairy cows was dedicated to a minimum number of authors. Pregnant cows are likely to be problematic; therefore, the study of their behaviour is absent. However, they are always present in the herd, so we wanted to include this in our observations. According to our empirical experience, we have compiled a design experiment.

The mixing of new cows after relocation can create social tension [12]. The previous research has considered only the grouping of similar cattle, for example, as they change between feeding groups. Yet the cows usually end up in approximately the same relative position in the dominance hierarchy as previously, with no loss of position as a result of the move [12,13]. However, there is little evidence to support the conclusion that pregnant animals suffer in terms of well-being when forced to compete with open cows. According to the majority of authors, the most important factors in determining social position are age and live body weight. However, it is almost impossible to control for other factors, as age is often associated with seniority in the group, weight and experience [38,48].

With advancing gestation, dairy cows become slower. For most pregnant cows, sexual activity is not clear but some accompany open cows in heat and can even jump on them. They do not try to improve their social order, avoid conflicts and tend to seek contact in the form of social contacts, such as sniffing and licking. Other cows respect their condition. However, we cannot say that they are strictly submissive. Rather, they only protect the fetus and therefore do not enter into conflict. Pregnant cows are, therefore, often considered to suffer the most in new social environments [38,60].

Moreover, the effects of combining stressors may cause aggravated welfare, especially the adaptation to a distinct type of housing [34,48,51]. Cows in the present study decreased their lying times directly after removing on the first day but partially returned to usual levels the following day. Overcrowding could contribute to a high variation in individual lying bouts [34,59]. A better understanding of the components associated with the relocation process is needed to identify the major stressors and at what point multiple stressors begin to impact endocrine, immunological and metabolic functions in a manner that jeopardizes the health and well-being of cattle [15,43].

## 5. Conclusions

The observed cows generally lay down up to ten hours after moving, milk production decreased significantly immediately after relocation and return to baseline occurred after 14 days.

The results of this study suggest that removing cows from the tie-stall barn with a pipeline milking system into the barn with free-stall housing and a milking parlour caused a decline in cows’ milk production. However, when the cows are moved to a better environment, they rapidly adapt to change.
